# Elephant space use and habitat selection change across drought timescales

**DOI:** 10.1111/1365-2656.70289

**Published:** 2026-06-22

**Authors:** Irene Bouwman, Tom Claassen, Michael J. Chase, Kelly Landen, Mark A. J. Huijbregts, Marlee A. Tucker

**Affiliations:** ^1^ Department of Environmental Science, Radboud Institute for Biological and Environmental Sciences Radboud University Nijmegen The Netherlands; ^2^ Department of Data Science, Institute for Computing and Information Sciences Radboud University Nijmegen The Netherlands; ^3^ Elephants without Borders Kasane Botswana

**Keywords:** climate change, habitat selection, home range, *Loxodonta africana*, movement ecology, Standardized Precipitation‐Evapotranspiration Index

## Abstract

1. Droughts are globally increasing in duration, frequency and intensity, thereby endangering the survival of wildlife species. Understanding how animals behave during drought is essential to develop conservation measures that aid wildlife survival and prevent human–wildlife conflict in a changing climate. Yet, little is known about animal behaviour during drought. Specifically, existing work on animal behaviour and drought typically does not account for the timescale and the intensity of the drought.
2. We use 19 years of GPS data from the African elephant (*Loxodonta africana*), a species at risk from drought, collected in Southern Africa, where droughts are increasingly common. We study if there is a connection (i) between drought and the amount of space elephants use and (ii) between drought and elephant habitat selection. We examine drought conditions using the Standardized Precipitation‐Evapotranspiration Index (SPEI) at a 1‐month, a 3‐month, and a 12‐month timescale.
3. We found that elephants had smaller home ranges and movement distances when their environment became drier at a 1‐month timescale. Importantly, SPEI at a 1‐month timescale was a better predictor of elephant home range size than precipitation and NDVI, suggesting that drought conditions may affect elephant movement through more ways than a lack of precipitation or vegetation greenness. In addition, we found that elephant habitat selection changed as a function of drought conditions at all three timescales, but the direction of the change often depended on the timescale of the drought. We found that elephants were closer to inland water and in areas with lower human population density during drought at a 1‐month timescale, but farther from inland water and in areas with higher human population density when drought lasted longer.
4. Overall, our results show that drought conditions, as measured by SPEI, predict elephant movement behaviour. We stress the importance of (i) using a drought index rather than only precipitation levels to examine drought conditions and (ii) considering the timescale at which drought occurs. These insights could help to better understand how animals behave during drought and to assess the risk of human–wildlife conflict across species, regions and drought timescales.

## Graphical abstract

Drought threatens wildlife worldwide, but little is known about wildlife behaviour during drought. This study combines 19 years of elephant GPS data with drought conditions at three timescales. Key findings include that elephants (i) move less during drought at a 1‐month timescale and (ii) change habitat selection across drought timescales.

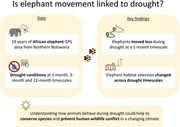

## 1 INTRODUCTION

Droughts are globally increasing in frequency, duration and intensity (Trenberth et al., 2014; Xu et al., 2019). Drought endangers the survival of wildlife, because animals can die of dehydration, starvation, wildfire or human–wildlife conflict (Abrahms et al., 2023; Wato et al., 2016). Survival of wildlife in an increasingly dry climate depends on (i) the ability to behaviourally adapt to drought, and (ii) whether effective conservation measures are taken in case behavioural adaptation does not suffice. To develop conservation measures that aid wildlife survival and prevent human–wildlife conflict, it is important to understand if and how animals alter their behaviour during drought.

Although it is well established that drought threatens wildlife survival, little is known about the connection between drought and wildlife behaviour. In particular, existing research on wildlife behaviour in relation to drought typically does not consider the timescale and the intensity of the drought (Slette et al., 2019; but see West et al., 2024). Yet, the effect of drought on resource availability strongly depends on the timescale at which the drought occurs. Drought starts upon a prolonged lack of rainfall (meteorological drought), but as drought persists, vegetation declines and crop yields drop (agricultural drought), and ultimately water levels of lakes, rivers and groundwater decline (hydrological drought) (Van Loon, 2015). The intensity indicates whether drought conditions are within normal climate variability, such as during annually recurring dry seasons, or fall outside of normal climate variability (Slette et al., 2019). This difference is important since animals may be prepared for predictable dry seasons but not for the increasingly frequent, long and intense droughts expected in the coming decades (Slette et al., 2019;Trenberth et al., 2014; Xu et al., 2019). For example, droughts or subsequent wildfires could cause animals to look for shelter in human‐dominated areas, potentially leading to human–wildlife conflict (Abrahms et al., 2023). At larger spatio‐temporal scales, climate change may disrupt long‐distance movements such as seasonal migration, because the timing of resource availability along the migration route shifts (Kubelka et al., 2022). Thus, accounting for drought timescale and intensity is expected to help in understanding how animals behave in a changing climate.

The African elephant (*Loxodonta africana*) is an ecosystem engineer known for its large‐scale movements that allow seed dispersal over long distances (Coverdale et al., 2016; Poulsen et al., 2021). Yet, the African elephant is endangered because of climate change and conflict with humans (Black et al., 2024). In Southern Africa, which has the largest elephant population in the world, severe droughts occurred in 2023 and 2024 and will become more frequent over the 21st century (Black et al., 2024; Schlossberg & Chase, 2024; UNCCD, 2025; Xu et al., 2019). Drought causes elephant mortality because elephants starve, dehydrate or get stuck in muddy waters (UNCCD, 2025; Wato et al., 2016). Moreover, drought increases the risk of human–elephant conflict, in the first place because humans and elephants compete for limited resources (Abrahms et al., 2023; Mariki et al., 2015), but also because measures to prevent human–elephant conflict may be less effective during drought (King et al., 2024).

Previous work on elephant behaviour and drought conditions has mainly focused on differences between annual wet and dry seasons with fixed start and end dates. During the wet season, precipitation is higher and vegetation is greener compared to the dry season. In general, elephants have larger home ranges during the wet season than during the dry season (Makati et al., 2025; Thomas et al., 2012), and vegetation greenness and precipitation have been linked to larger home ranges (Beirne et al., 2021; Benitez et al., 2022; Wall et al., 2021). However, as seasons become more unpredictable due to climate change, a classification between a wet and a dry season with fixed start and end dates may not reflect the meteorological conditions at the study site (Mulder et al., 2024). Second, drought conditions are not only driven by precipitation, but also by temperature. Indeed, global warming is expected to speed up the onset and increase the duration and intensity of droughts (Trenberth et al., 2014). Finally, it is not clear whether elephant behaviour during predictable, annually recurring dry seasons reflects how elephants behave during climate change‐induced droughts that exceed the drought conditions of annual dry seasons in both duration and intensity (Black et al., 2024; Slette et al., 2019).

Here, we study the extent to which drought conditions at three different timescales are associated with three aspects of elephant movement: home range size, movement distance and habitat selection. To that end, we use 19 years of African elephant GPS data from Southern Africa. We examine drought conditions using the Standardized Precipitation‐Evapotranspiration Index (SPEI) (Vicente‐Serrano et al., 2010). The SPEI is a standardized metric that measures the current balance between precipitation and evapotranspiration, which is affected by temperature, relative to the long‐term balance. The SPEI quantifies the intensity of drought conditions ranging from extreme drought (SPEI ≤ −2) to extremely wet (SPEI ≥ 2). We use the SPEI accumulated over 1 month (SPEI01), 3 months (SPEI03) and 12 months (SPEI12), corresponding to meteorological, agricultural and hydrological drought, respectively (Liu et al., 2024; Wang et al., 2021). Table 1 lists the expected relationships between drought and changes in elephant home range size, movement distance and habitat selection.

**TABLE 1:** Hypothesized changes in elephant home range size, movement distance and habitat selection during drought.

Variable	Expected response	Hypothesis
Home range size Movement distance	Smaller home ranges and movement distances	Elephants have smaller home ranges during the dry season (Makati et al., 2025; Thomas et al., 2012). Since restricting movement may be a strategy to save energy when the environment becomes drier, elephant home range sizes as well as movement distances decline during drought
Distance to inland water Distance to artificial waterholes	Lower selection for areas farther from inland waters and artificial waterholes	Elephants need to drink water regularly, with a maximum of 3 or 4 days between two drinking events (Chamaillé‐Jammes et al., 2013). Elephants are closer to permanent water sources in the dry period compared to the wet period (Bastille‐Rousseau et al., 2020). Water sources in the study area consist of inland waters, artificial waterholes and rain‐filled pans (Chase, 2017). During drought, rain‐filled water sources may dry up, and therefore, elephants stay closer to inland waters and artificial waterholes
Precipitation	Higher selection for areas with more precipitation	Elephants select more strongly for precipitation when it is drier, because the sites in the home range that receive more rain may have more fruits (Danquah & Oppong, 2007) and water in rain‐filled pans available
NDVI	Higher selection for areas with higher NDVI	Elephants in general select for areas with higher NDVI (Branco et al., 2019; Tsalyuk et al., 2019). Elephants select more strongly for NDVI when it is drier, because sites with sufficient vegetation quality become more sparse and therefore the need to select for areas where NDVI is relatively high becomes larger
Tree cover	Higher selection for areas with more tree cover	During drought, elephants switch to a diet with more woody vegetation (Abraham et al., 2019). Hence, elephants select more strongly for tree cover when the environment becomes drier
Slope	(a) Lower selection for slopes (b) Higher selection for slopes	Elephants avoid areas where moving is energetically costly, including slopes (Bastille‐Rousseau et al., 2020; Berti et al., 2025). We considered two hypotheses: (a) As resources become sparse during drought, it is important for elephants to conserve energy. Therefore, selection for slopes declines during drought. (b) Resource depletion may drive elephants to visit less favourable areas in search of water and food. Therefore, selection for slopes increases during drought
Croplands Human population density	Higher selection for croplands and areas with a higher human population density	During drought, elephants enter human‐dominated areas in search of resources (Abrahms et al., 2023; Mariki et al., 2015). Thus, selection for croplands and human‐populated areas increases during drought

## 2 MATERIALS AND METHODS

### 2.1 Study area

**FIGURE 1:**
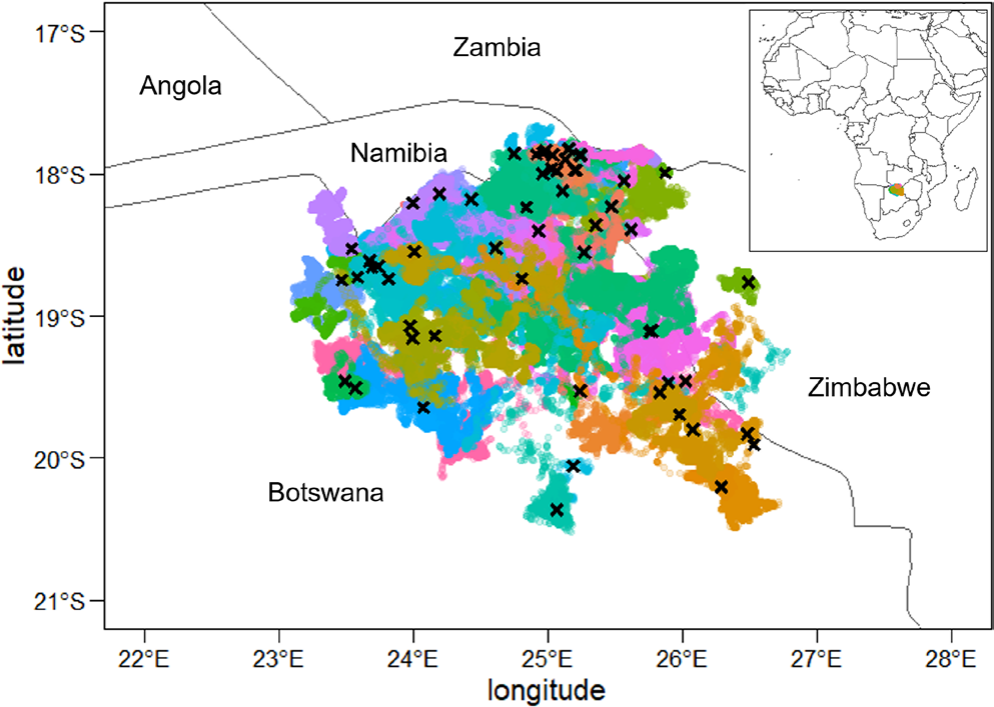
Map of the study area with GPS location records of 57 African elephants collected from 2004 to 2022. Colours indicate different elephants. The total number of GPS location records is 504,224. Black crosses indicate the first GPS record of each elephant.

African elephants included in this study were tagged in and around Chobe National Park, Botswana (Figure 1). The Chobe River and the Linyanti River at the border between Botswana and Namibia, and the Zambezi River at the border between Zambia and Zimbabwe are the largest inland waters in the study area (Chase, 2017). Other water sources in the study area are smaller rivers surrounded by wetlands that seasonally flood, water pans filled by rainfall and artificial waterholes (Chase, 2017).

The study area mainly consists of large protected areas and sparsely developed areas. Croplands are dispersed over the study area, but primarily found along the Chobe river. Human settlements are also mainly located along the Chobe River. The largest human settlement in the study area is Kasane, with an estimated population size of 9095 inhabitants in 2022 (Statistics Botswana, 2022). Notably, since the Chobe National Park borders both the Chobe River and the Linyanti River, elephants can access the rivers without crossing croplands or human settlements.

**FIGURE 2:**
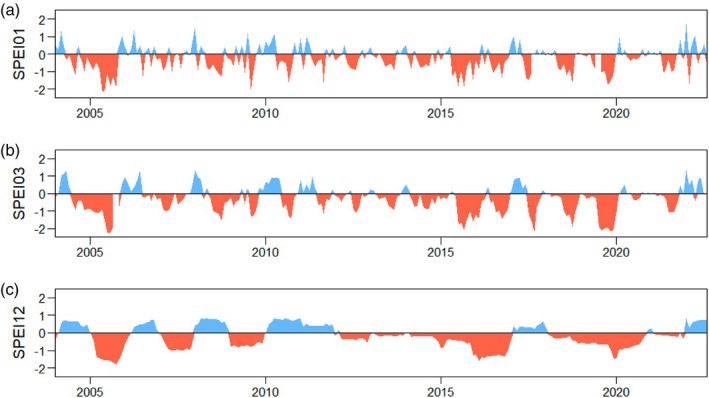
Mean Standardized Precipitation‐Evapotranspiration Index (SPEI) in the study area. The SPEI is accumulated over the past month (SPEI01, a), the past 3 months (SPEI03, b) or the past 12 months (SPEI12, c). Negative values of SPEI indicate an excess of evapotranspiration relative to precipitation compared to the long‐term average, so a lower SPEI value means that the study area is drier. Study area: 100% minimum convex polygon around all GPS locations of all elephants in the study.

Botswana has a dry season characterized by low rainfall from May to October and a wet season from November to April. During the study period (2004–2022), SPEI01 was on average the lowest in the middle of the dry season, in August (mean SPEI01 = −0.92, SD = 0.56), and the highest in April, at the end of the wet season (mean SPEI01 = 0.35, SD = 0.63). SPEI12 was below −1.5 in 2005, 2016 and 2019, indicating severe to extreme drought (Figure 2) (Tan et al., 2015).

### 2.2 Elephant GPS data

GPS locations of 57 African elephants were collected in the North of Botswana from 2004 to 2022 by Elephants Without Borders (Figure 1). The Department of Wildlife and National Parks and the Ministry of Environment and Tourism of Botswana gave permission to conduct the study under the permit numbers ‘OP 46/1 LXXXV (89)’, ‘EWT 8/36/4 XXIX (41)’, ‘ENTC 8/36/4 LVIII (173)’. We removed points with a speed above 7 km/h to exclude GPS tracking errors (Wall et al., 2013), and we removed the first 7 days of each track to exclude behavioural alterations following the GPS collaring event (Stiegler et al., 2024). The final dataset contained 504,224 GPS location records of 57 elephants and 1025 elephant‐year‐month combinations, with more than 25 days of data per individual per month and a maximum of 8 h between two subsequent location records in a month (Figure S1).

### 2.3 Home range size and movement distance

We estimated monthly home ranges for each individual by calculating a 95% minimum convex polygon (MCP) around the individual's monthly GPS records using the *adehabitatHR* package (Calenge, 2011) in Rstudio 4.3.3. To obtain the monthly distance moved, we calculated the distance between each first GPS location of a day and the first GPS location of the previous day using the *raster* R package (Hijmans et al., 2013) and took the sum for each month.

To assess if there is a connection between drought conditions and elephant movement, we extracted rasters of environmental variables using Google Earth Engine accessed via the *ee* package (Gorelick et al., 2017) in Python 3.12.5 (Table 2). We extracted rasters with SPEI accumulated over 1 month (SPEI01), 3 months (SPEI03) and 12 months (SPEI12) from SPEIbase v2.10 (Beguería et al., 2024; Vicente‐Serrano et al., 2010). We annotated the 95% MCP monthly home range of each individual with the mean SPEI01, SPEI03 and SPEI12 in the 95% MCP home range during the month. In addition, we included human disturbance as a predictor because elephants have smaller home ranges in areas with more human disturbance (Beirne et al., 2021; Wall et al., 2021). To account for human disturbance, we used the Human Impact Index (HII), which measures human disturbance based on the covariates human population density, built infrastructure, land use, power consumption and accessibility (Sanderson et al., 2002). We extracted rasters of the annual HII in the study area and calculated the mean HII in each 95% MCP monthly home range. We also included monthly cumulative precipitation and the Normalized Difference Vegetation Index (NDVI), because precipitation and NDVI are associated with an increase in elephant home range size (Beirne et al., 2021; Benitez et al., 2022; Wall et al., 2021). We extracted rasters with monthly cumulative precipitation from the Climate Hazards Center InfraRed Precipitation with Station data (CHIRPS) dataset and calculated the monthly cumulative precipitation in each 95% MCP monthly home range (Funk et al., 2015). We extracted rasters of monthly mean NDVI using the MOD13A1.061 Terra Vegetation Indices dataset (Didan, 2021) and calculated the monthly mean NDVI in each 95% MCP monthly home range. Finally, we included temperature as a predictor, because temperature influences drought conditions (Vicente‐Serrano et al., 2010). We extracted rasters with the monthly mean of the maximum daily temperature for each raster cell using the CPC Global Unified Temperature dataset from the NOAA Physical Sciences Laboratory (https://psl.noaa.gov) and calculated the mean value in each 95% MCP monthly home range.

**TABLE 2:** Environmental data used in the study. For variables that do not cover the entire timespan of the study, the timespan is indicated between brackets.

Variable	Source	Temporal resolution	Spatial resolution
Human Impact Index	Wildlife Conservation Society (Sanderson et al., 2002; https://wcshumanfootprint.org/)	1 year (2004–2020)	300 m
NDVI	MOD13A1.061 Terra Vegetation Indices (Didan, 2021)	16 days	500 m
Precipitation	Climate Hazards Center Infrared Precipitation with Stations (CHIRPS) (Funk et al., 2015)	1 day	0.05° (≈5566 m)
Temperature	CPC Global Unified Temperature, NOAA Physical Sciences Laboratory (https://psl.noaa.gov)	1 day	55,500 m
SPEI	SPEIbase v2.10 (Beguería et al., 2024; Vicente‐Serrano et al., 2010)	1 month	0.5° (≈55,500 m)
Elevation	NASA Digital Elevation Model (NASADEM) (Crippen et al., 2016)	NA	30 m
Tree cover	MOD44B.061 Terra MODIS Vegetation Continuous Fields (DiMiceli et al., 2022)	1 year (2004–2020)	250 m
Population density	Wildlife Conservation Society (Sanderson et al., 2002; https://wcshumanfootprint.org/)	1 year (2004–2020)	300 m
Distance to cultivated area	WorldPop (Woods et al., 2025)	1 year	3 arc seconds (≈100 m)
Distance to inland water	WorldPop (Woods et al., 2025)	NA	3 arc seconds (≈100 m)
Waterhole locations	Elephants Without Borders (personal communication)	NA	NA

*Note*: Spatial points were annotated with environmental data of the closest time point.

Abbreviation: NA, not applicable.

We assessed if SPEI was a predictor of monthly home range size and movement distance in multivariate linear mixed models using the *glmmTMB* R package (Brooks et al., 2017). We fit a baseline model to predict monthly home range with HII, NDVI, precipitation and temperature as environmental predictors and elephant ID as a random effect to account for non‐independence of observations from the same individual. Next, we extended the baseline model by including all combinations of SPEI01, SPEI03 and SPEI12 as predictors, leading to eight models in total. We repeated the modelling approach to predict monthly distance moved. We log_10_‐transformed home range size and movement distance to meet the assumption that residuals are normally distributed, which we checked by inspecting Q‐Q plots. Pearson and Spearman correlation coefficients between predictors were below 0.7 in each model (Figure S2). We standardized the predictor variables prior to the analysis by subtracting the mean and dividing by the standard deviation. We used the Akaike information criterion (AIC) to identify which of the tested models performed best. To check for robustness of our results, we also fit univariate linear mixed models for each of the predictors in our best model separately, with ID as a random effect.

### 2.4 Resource selection

We used resource selection functions to assess if elephant habitat preferences change as a function of SPEI. To that end, we extracted rasters of the environmental variables listed in Table 1 to annotate each of the GPS points in our data with the value of the environmental variable at the closest time point available (Table 2). We used Google Earth Engine accessed via the *ee* package (Gorelick et al., 2017) in Python 3.12.5 to extract rasters unless stated otherwise. We downloaded rasters with the distance to inland water from WorldPop (Woods et al., 2025). Spatial locations of artificial waterholes were provided by Elephants Without Borders, and we created a raster with the distance to the nearest waterhole for each GPS point using the *terra* package in Rstudio 4.3.3 (Hijmans et al., 2025). We did not have data on the amount of water available in inland waters and artificial waterholes. We extracted rasters with monthly cumulative precipitation from the Climate Hazards Center InfraRed Precipitation with Station data (CHIRPS) dataset (Funk et al., 2015) and rasters with monthly mean NDVI from the MOD13A1.061 Terra Vegetation Indices dataset (Didan, 2021). We extracted rasters with the percentage of tree cover per raster cell from the MOD44B.061 Terra MODIS Vegetation Continuous Fields dataset (DiMiceli et al., 2022). We extracted an elevation raster using the NASA Digital Elevation Model (NASADEM) (Crippen et al., 2016) and converted it to a slope raster using the *Terrain.slope* function in Google Earth Engine. We used the human population density covariate layer of the HII to extract rasters with human population density (Sanderson et al., 2002). We downloaded rasters with the distance to cultivated area from WorldPop (Woods et al., 2025). We binarized the rasters with distance to cultivated area by assigning a value of 0 to non‐cultivated raster cells (distance to cultivated area >0) and a value of 1 to cultivated raster cells. For 0.3% of the GPS points, the value of at least one of the environmental covariates was missing, and we omitted these GPS points from the resource selection analysis.

Resource selection functions compare environmental variables at visited GPS locations and available locations randomly sampled from the individual's monthly home range. We sampled 30 random points per visited point from each 95% MCP monthly home range and annotated the randomly sampled points with the same environmental variables as the visited points following the procedure described above. We omitted random points with missing values for at least one of the environmental covariates (0.3% of the total number of random points). Next, we fit generalized linear mixed models with a binomial distribution using the *glmmTMB* R package (Brooks et al., 2017) to predict visited GPS locations (1) and available locations randomly sampled from the individual's 95% MCP monthly home range (0) from the environmental variables (Table 1). To test whether selection for environmental variables changed during drought, we added an interaction term between each environmental variable and the mean SPEI in the individual's 95% MCP monthly home range that we extracted from SPEIbase v2.10 (Beguería et al., 2024; Vicente‐Serrano et al., 2010). We fit three separate models with interactions between predictors and (i) SPEI01, (ii) SPEI03 and (iii) SPEI12. Pearson and Spearman correlation coefficients between predictor variables were all below 0.7 (Figure S3). All predictors were standardized by subtracting the mean and dividing by the standard deviation prior to the analyses. We included elephant ID as a random effect to account for non‐independence of data points from the same individual. We repeated the resource selection analysis with 1, 5, 10 and 20 random points per visited point to confirm that model coefficients had converged at 30 points (Fieberg et al., 2021).

## 3 RESULTS

### 3.1 Home range size and movement distance

The median size of elephant monthly home ranges was 2045 km^2^ (25th percentile = 896 km^2^, 75th percentile = 5042 km^2^), and the median monthly movement distance was 142 km (25th percentile = 94 km, 75th percentile = 507 km).

**FIGURE 3:**
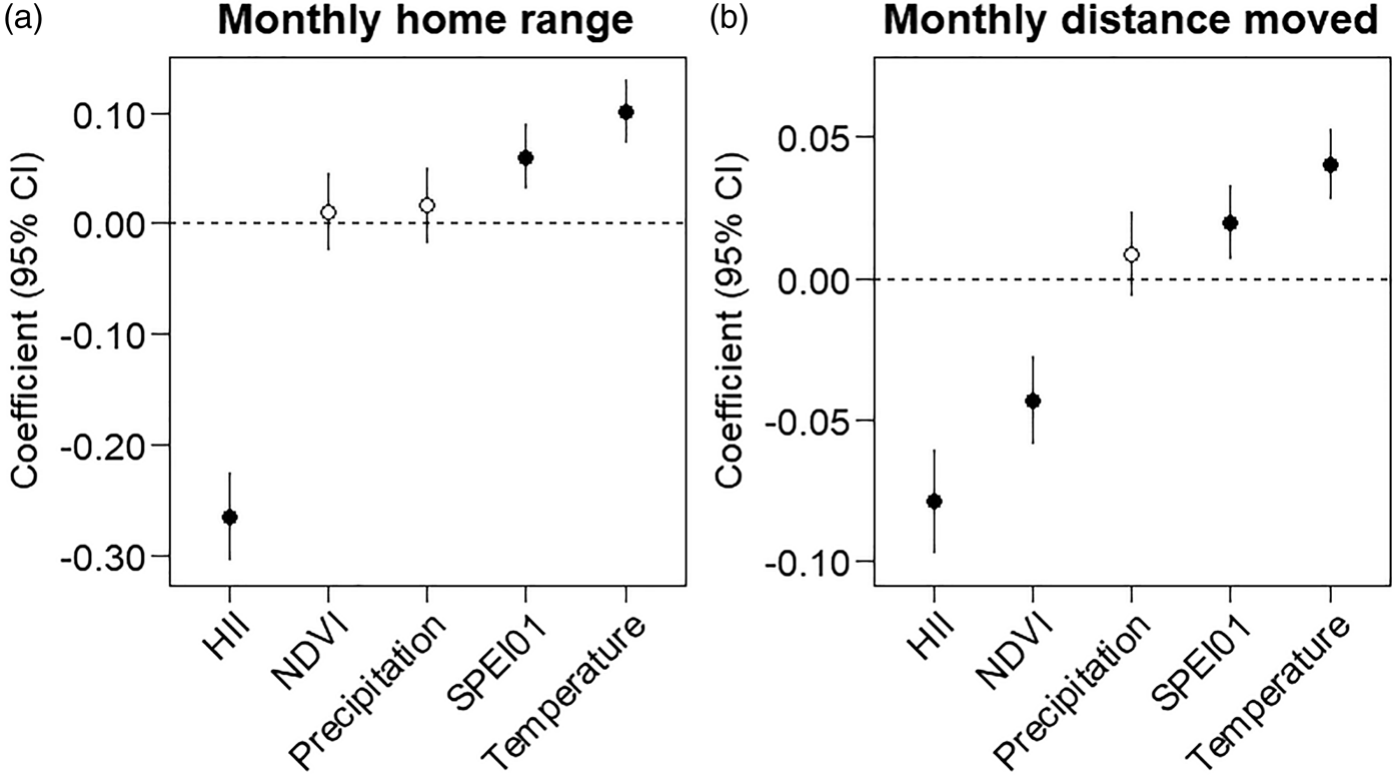
Model coefficients and 95% confidence intervals for predictors of elephant monthly home range (a), and distance moved (b). HII, Human Impact Index; NDVI, Normalized Difference Vegetation Index; SPEI01, Standardized Precipitation‐Evapotranspiration Index at a 1‐month timescale. Higher HII, NDVI and SPEI values mean that the home range is more human‐impacted, greener and wetter, respectively. Open circles indicate estimates where confidence intervals overlap zero.

We fit a multivariate model with HII, NDVI, precipitation and temperature and extended it with all combinations of SPEI01, SPEI03 and SPEI12 as predictors. The best model we tested included SPEI01 but not SPEI03 and SPEI12 (AIC; Table S1). Notably, temperature, HII and SPEI01 were significant predictors of home range size in the best tested model, but precipitation and NDVI were not (Table S2; Figure 3a). Home range size, on average, decreased by a factor of 2.5 from wet to dry conditions at a 1‐month timescale (SPEI01 range: −3.0 – 2.2; Figure S4).

Monthly movement distance was negatively associated with HII and NDVI, not associated with precipitation, and positively associated with SPEI01 and temperature in the best model we tested (AIC; Table S1; Table S2; Figure 3b). Monthly movement distance, on average, decreased by a factor of 1.4 from wet to dry conditions at a 1‐month timescale (SPEI01 range: −3.0 – 2.2; Figure S4).

### 3.2 Resource selection

**FIGURE 4:**
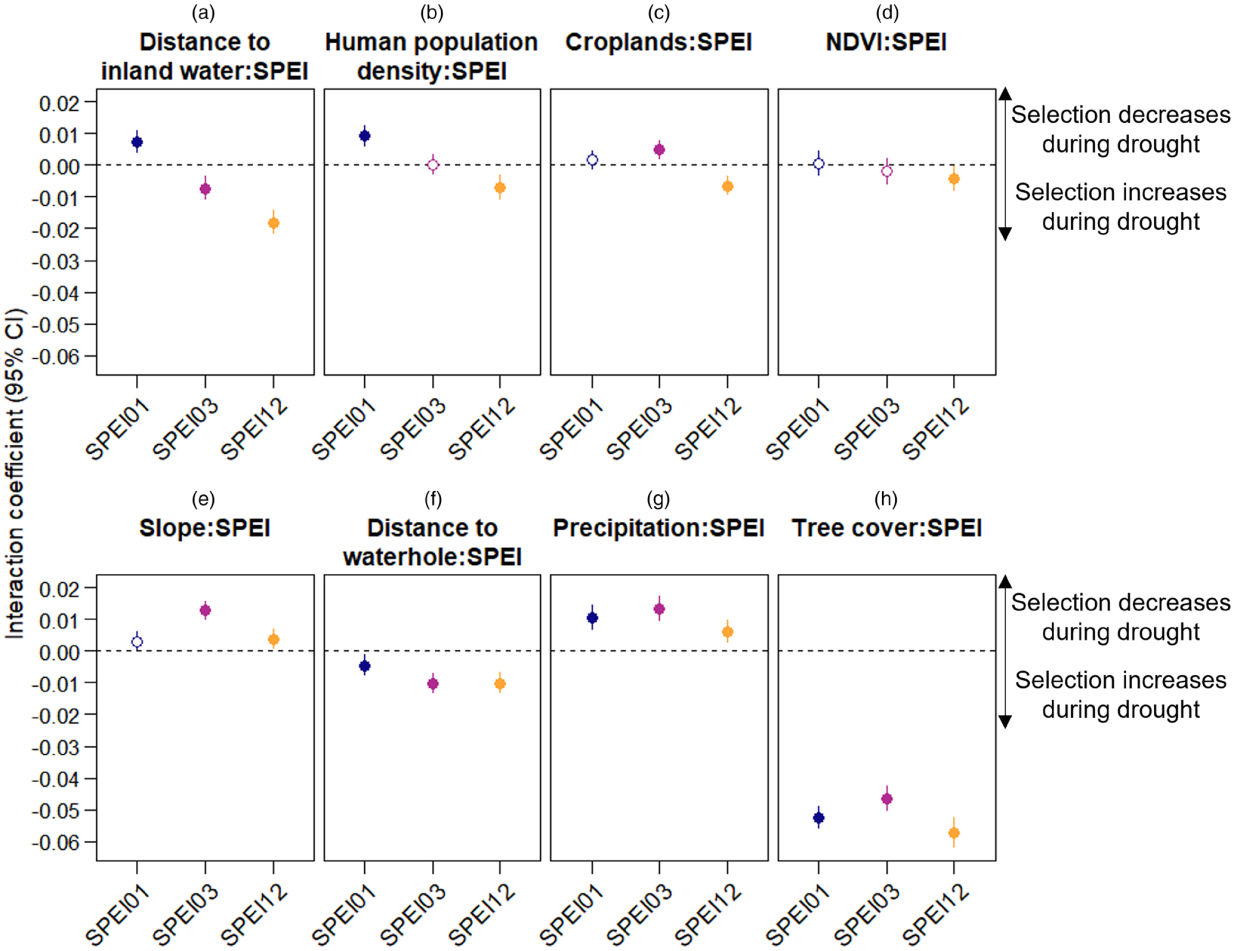
Interaction coefficients with 95% confidence intervals for the interactions between predictors of elephant resource selection (a–h) and the Standardized Precipitation‐Evapotranspiration Index (SPEI) at a 1‐month (SPEI01, blue), 3‐month (SPEI03, purple) and 12‐month timescale (SPEI12, yellow). Open circles indicate estimates where confidence intervals overlap zero. Note that a negative interaction coefficient means that the selection coefficient of the covariate is higher when the home range is drier.

We found interactions between elephant resource selection and SPEI at all three drought timescales (Figure 4; Tables S3–S5). Since a lower SPEI indicates drier conditions, a positive interaction between SPEI and a predictor means that the selection coefficient of the predictor decreases when the home range becomes drier. For distance to inland water, human population density, croplands, NDVI and slope, the direction of the change in selection during drought depended on the drought timescale. Selection for distance to inland water positively interacted with SPEI01, indicating that elephants were closer to water during short‐term drought (Figure 4a, blue point). However, SPEI03 and SPEI12 negatively interacted with distance to inland water, indicating that elephants selected locations farther from inland water when drought lasted longer (Figure 4a, purple and yellow points). Similarly, elephants selected sites with lower population density during drought at a 1‐month timescale, but selected sites with a higher population density during drought at a 12‐month timescale (Figure 4b). We did not find an interaction between cropland selection and SPEI01 (Figure 4c, blue point). We found a small positive interaction between selection for croplands and SPEI03, indicating that the selection coefficient for croplands declined during drought at a 3‐month timescale (Figure 4c, purple point). At a 12‐month timescale, we found a negative interaction between selection for croplands and SPEI, indicating that elephants were more often in croplands during long‐term drought (Figure 4c, yellow point). We found a small negative interaction between NDVI and SPEI12, suggesting that elephants selected more strongly for NDVI during long‐term drought, but selection for NDVI did not interact with SPEI01 and SPEI03 (Figure 4d). We did not find an interaction between selection for slopes and SPEI01 (Figure 4e, blue point). However, we did find positive interactions between SPEI03 and slope and between SPEI12 and slope, indicating that the selection coefficient for slopes declined during drought at a 3‐month and a 12‐month timescale (Figure 4e, purple and yellow points).

For distance to artificial waterholes, precipitation and tree cover, the change in selection during drought did not depend on the drought timescale. Selection for distance to artificial waterholes interacted negatively with SPEI at all three drought timescales, indicating that elephants were farther from artificial waterholes during drought (Figure 4f). We found positive interactions between precipitation and SPEI at all three timescales, suggesting that elephants' selection for precipitation declined during drought (Figure 4g). Selection for tree cover interacted negatively with SPEI regardless of the drought timescale, indicating that elephants selected for more tree‐covered areas during drought (Figure 4h).

## 4 DISCUSSION

Predicting how climate change affects animal behaviour is challenging, but necessary to conserve species and prevent human–wildlife conflicts (Buchholz et al., 2019). In this study, we assessed how drought at three different timescales is associated with the movement behaviour of the African elephant, whose habitat will face droughts of increasing duration, frequency and intensity over the coming decades due to climate change (Black et al., 2024; UNCCD, 2025; Xu et al., 2019). We found that elephant home ranges and movement distances declined as the environment became drier at a 1‐month timescale. Notably, drought conditions at a 1‐month timescale were a better predictor of elephant home range size than precipitation and NDVI. In addition, we found that elephant habitat selection changed during drought, and for most covariates, the direction of the association between drought and habitat selection depended on the timescale of the drought. For example, we found that elephants selected sites closer to water and with lower human population density during short‐term drought (timescale: 1 month), but selected sites farther from water and with higher human population density as drought lasted longer (timescale: 12 months).

NDVI and precipitation have repeatedly been identified as predictors and interpreted as drivers of elephant home range size (Beirne et al., 2021; Benitez et al., 2022; Wall et al., 2021), and NDVI is a well‐established predictor and presumed driver of home range size across mammal species (Broekman et al., 2024). However, neither NDVI nor precipitation were significant predictors of home range size in the best‐supported model tested here, while SPEI01 was. Interestingly, this suggests that elephants respond to other characteristics of drought than a lack of rainfall or vegetation browning. Future research could explore if there is a connection between elephant movement and soil characteristics, such as nutrient levels and soil moisture, because elephants not only feed on vegetation but also extract nutrients from soil (geophagy) (Holdø et al., 2002; Klaus et al., 1998). This could help to understand which aspects of drought drive a decline in elephant home range size and movement distance.

At all three drought timescales, we found that elephants changed their habitat selection as their environment became drier. For some covariates, selection consistently increased or decreased during drought regardless of the drought timescale. As expected, we found an increase in selection for tree‐covered areas during drought at all three timescales. This is consistent with prior research reporting that elephants shift to a diet with more woody vegetation during drought (Abraham et al., 2019) and that tree damage by elephants increases during drought (Thornley et al., 2020). In addition, elephants may search for shade in tree‐covered areas to cope with high temperatures during drought (Kinahan et al., 2007).

Contrary to our predictions, elephants were farther from artificial waterholes and selected less for precipitation when their environment became drier at all three timescales. Waterholes are spread out across the study area and thereby cover a large area available for foraging. However, competition at waterholes may increase during drought, which may restrict the access of cows and calves as they are outcompeted by adult bulls (M.J.C., personal communication). Future research could explore if selection for artificial waterholes during drought depends on elephant sex or age. Moreover, waterholes can be dry or not working, and may therefore not be a reliable source of water during drought. Similarly, rain‐filled pans may dry up faster when the environment is drier, which might explain why selection for precipitation declines during drought. Finally, elephants may need to forage farther from waterholes during drought because the vegetation around waterholes is depleted (Thrash, 1998; Valls‐Fox et al., 2018).

For most environmental variables tested, the change in habitat selection during drought depended on the drought timescale. Importantly, this indicates that elephant behaviour during long‐term drought is not always an extrapolation of elephant behaviour during the short‐term dry conditions that may be common during annually recurring dry seasons. At the 1‐month timescale, elephants were closer to inland waters during drought, in line with our predictions and with previous research reporting that elephants are closer to permanent water sources during the dry season (Bastille‐Rousseau et al., 2020). In contrast, at the 3‐month and 12‐month timescales, elephants were farther from inland water when their environment became drier, possibly because vegetation around inland water sources was exhausted or the water sources dried up.

A limitation of our study is that it did not include data on the amount of water available in water bodies and artificial waterholes. During drought, water sources may dry up, which could explain why elephants were farther from artificial waterholes during drought at all three timescales and farther from inland water sources at drought timescales beyond 1 month. However, temporally explicit data on water availability in artificial waterholes may be difficult to obtain. As for inland waters, future research could explore surface water extent data to estimate the amount of inland water at a monthly or annual resolution (Pekel et al., 2016; Reiter et al., 2015). This could help to understand if the differences in elephant selection for inland water between short‐ and long‐term drought could be explained by the amount of water available in inland water sources.

During drought at a 1‐month timescale, elephants selected for areas with lower human population density, contrary to our predictions. However, elephants selected for areas with higher human population density during drought at a 12‐month timescale, consistent with an increased incidence of human–wildlife conflict during drought (Abrahms, 2021; Abrahms et al., 2023). Although selection for NDVI, croplands and slopes were not related to drought at a 1‐month timescale, elephants selected more strongly for NDVI, croplands and areas with less steep slopes during drought at a 12‐month timescale. As vegetation declines during drought, the need for elephants to select for NDVI may become higher, and in the lack of natural vegetation elephants may forage on crops (Abrahms et al., 2023; Mariki et al., 2015). Moreover, elephants may avoid slopes during long‐term drought to conserve energy when resources are scarce.

Taken together, our results point to a timescale‐dependent response of elephants to drought. At the onset of a drought, elephants might limit their movement to gather around inland water sources, but as drought lasts and vegetation around inland waters may be exhausted, elephants could prefer to move to human‐dominated areas in search of resources. Future research could investigate whether these findings expand to elephants living in different climate conditions and landscape configurations. Notably, elephant populations in both dry and wet savannahs have smaller home ranges in the dry season compared to the wet season (Young et al., 2009), which might suggest that elephants respond to drier‐than‐average conditions by reducing their home range size regardless of the local climate.

Although it is well‐established that human activity globally affects animal movement, the variables used to measure human activity typically do not account for human‐induced climate change (Broekman et al., 2024; Tucker et al., 2018). Climate change is a global threat to biodiversity, but there are still many open questions regarding the effect of climate change on animal behaviour (Buchholz et al., 2019). Here, we found that African elephants in Botswana reduced their home range size upon drought at a 1‐month timescale. In contrast, West et al. (2024) conducted a study in the same study area and found that African wild dogs expanded their home range upon drought at a 3‐month timescale, and leopards and lions expanded their home range upon drought at a 12‐month timescale. This indicates that mammal species in the same environment exposed to the same climate conditions may nonetheless respond differently to drought. Hence, future studies could investigate if connections between home range size and drought can be explained by species traits, such as diet. Changes in home range sizes could affect ecosystem processes such as species interactions and seed dispersal (Doherty et al., 2021; Tucker et al., 2018), and thereby act as a pathway through which climate change affects ecosystem functioning.

Finally, drought is a global phenomenon that affects species across taxa and across regions (McTigue et al., 2026). Drought is expected to increase human–wildlife conflict worldwide, ranging from crocodile attacks on livestock in South Sudan to crop raiding by Baird's tapirs in Mexico (Abrahms et al., 2023; Benansio et al., 2022; Pérez‐Flores et al., 2021). Hypothetically, our finding that elephants were more often in areas with higher human population density during long‐term drought might apply to other species. To test this, future research could assess the connection between drought and use of human‐populated areas for species across the globe. Importantly, our study demonstrates that (i) using a drought index rather than only precipitation levels, and (ii) examining drought across timescales can help to understand animal behaviour during drought. Future research could build on these insights by mapping patterns in wildlife behaviour during drought across species, regions and drought timescales, which would likely require long‐term GPS time series to capture variability in both short‐ and long‐term drought conditions. Overall, understanding how animals respond to drought may help to identify to what extent species behaviourally adapt to drought, and which conservation measures may be necessary to prevent human–wildlife conflict and conserve biodiversity in a changing climate.

## AUTHOR CONTRIBUTIONS

Irene Bouwman, Tom Claassen, Mark A.J. Huijbregts and Marlee A. Tucker conceived the ideas and designed the methodology. Michael J. Chase and Kelly Landen provided the GPS data. Irene Bouwman conducted the data analysis. Irene Bouwman, Tom Claassen, Mark A.J. Huijbregts and Marlee A. Tucker wrote the first draft of the manuscript. All authors contributed to the draft and gave final approval for publication.

## CONFLICT OF INTEREST STATEMENT

The authors declare no conflicts of interest.

## Supporting information


**Figure S1.** Timespan of GPS location records of 57 African elephants. Each row indicates a different elephant. Each time the color changes, there is a gap of more than 8 h between subsequent datapoints of the same individual.
**Figure S2.** Pearson correlation coefficients between predictor variables included in the linear mixed model to predict elephant monthly home range size and movement distance. SPEI01, SPEI03, SPEI12: Standardized Precipitation‐Evapotranspiration Index at a 1‐month, a 3‐month, and a 12‐month timescale, respectively. HII, Human Impact Index; NDVI, Normalized Difference Vegetation Index.
**Figure S3.** Pearson correlation coefficients between predictor variables used in resource selection analysis. NDVI: Normalized Difference Vegetation Index.
**Figure S4.** Model predictions (lines) with confidence intervals (shaded area) and observed monthly home ranges and movement distances (points) as a function of SPEI01. SPEI01: Standardized Precipitation‐Evapotranspiration Index at a 1‐month scale. The lower SPEI, the drier the home range. Both models included HII, NDVI, precipitation, temperature and SPEI01 as predictors, and elephant ID as a random effect. HII, NDVI, precipitation, and temperature are set to the mean value in these plots. *N* = 1025 monthly home ranges.
**Table S1.** Performance of multivariate linear mixed models to predict monthly home range size and distance moved. The baseline model has temperature, precipitation, NDVI and HII as predictors and individual ID as a random effect. Models are ordered from low to high AIC per response variable. *N* = 1025 elephant‐year‐month combinations.
**Table S2.** Model output for linear mixed models to predict monthly home range size and distance moved. Model output is shown for univariate models per predictor and for the best‐supported multivariate model. All models included individual ID as a random effect. Bold text indicates predictors that were significant in univariate but not in multivariate models. *N* = 1025 elephant‐year‐month combinations.
**Table S3.** Model output for a resource selection function (RSF) with interactions between environmental covariates and SPEI01. This model included elephant ID as a random effect. *N* = 57 elephants.
**Table S4.** Model output for a resource selection function (RSF) with interactions between environmental covariates and SPEI03. This model included elephant ID as a random effect. *N* = 57 elephants.
**Table S5.** Model output for a resource selection function (RSF) with interactions between environmental covariates and SPEI12. This model included elephant ID as a random effect. *N* = 57 elephants.

## Data Availability

Data available from the Dryad Digital Repository https://doi.org/10.5061/dryad.5mkkwh7m4 (Bouwman et al., 2026). The data contain (i) monthly home range sizes and movement distances of the individuals included in the study, annotated with environmental variables; and (ii) environmental variables at used locations (1) and locations available in the monthly home range (0) per individual per month.
