# Supplementary material for: Elephant space use and habitat selection change across drought timescales
Source: J Anim Ecol. 2026 Jun 22;95(8):1464–75. doi: 10.1111/1365-2656.70289 (PMC13439595; doi:10.1111/1365-2656.70289)
Supplement: Supplementary file 1 [file JANE-95-1464-s001.docx]

# 1 Supplementary information

**Figure S1: Timespan of GPS location records of 57 African elephants.** Each row indicates a different elephant. Each time the color changes, there is a gap of more than 8 hours between subsequent datapoints of the same individual.

**Figure S2: Pearson correlation coefficients between predictor variables included in the linear mixed model to predict elephant monthly home range size and movement distance.** SPEI01, SPEI03, SPEI12: Standardized Precipitation-Evapotranspiration Index at a 1-month, a 3-month, and a 12-month timescale, respectively. NDVI: Normalized Difference Vegetation Index. HII: Human Impact Index.

**Figure S3: Pearson correlation coefficients between predictor variables used in resource selection analysis.** NDVI: Normalized Difference Vegetation Index.

**Figure S4: Model predictions (lines) with confidence intervals (shaded area) and observed monthly home ranges and movement distances (points) as a function of SPEI01.** SPEI01: Standardized Precipitation-Evapotranspiration Index at a 1-month scale. The lower SPEI, the drier the home range. Both models included HII, NDVI, precipitation, temperature and SPEI01 as predictors, and elephant ID as a random effect. HII, NDVI, precipitation, and temperature are set to the mean value in these plots. *N* = 1,025 monthly home ranges.

**Table S1: Performance of multivariate linear mixed models to predict monthly home range size and distance moved.** The baseline model has temperature, precipitation, NDVI and HII as predictors and individual ID as a random effect. Models are ordered from low to high AIC per response variable. *N* = 1,025 elephant-year-month combinations.

| Response | Predictors | AIC | Marginal *R*^2^ | Conditional *R*^2^ |
| --- | --- | --- | --- | --- |
| Monthly home range | Base + SPEI01 | 1269.59 | 0.273 | 0.459 |
|  | Base + SPEI01 + SPEI12 | 1271.22 | 0.274 | 0.458 |
|  | Base + SPEI01 + SPEI03 | 1271.44 | 0.273 | 0.458 |
|  | Base + SPEI01 + SPEI03 + SPEI12 | 1273.17 | 0.274 | 0.457 |
|  | Base + SPEI03 | 1281.82 | 0.269 | 0.452 |
|  | Base | 1283.28 | 0.270 | 0.446 |
|  | Base + SPEI03 + SPEI12 | 1283.55 | 0.269 | 0.452 |
|  | Base + SPEI12 | 1285.27 | 0.270 | 0.446 |
|  |  |  |  |  |
| Monthly distance moved | Base + SPEI01 | -381.05 | 0.140 | 0.493 |
|  | Base + SPEI01 + SPEI03 | -380.18 | 0.140 | 0.490 |
|  | Base + SPEI01 + SPEI12 | -379.17 | 0.140 | 0.492 |
|  | Base + SPEI01 + SPEI03 + SPEI12 | -378.19 | 0.140 | 0.490 |
|  | Base | -374.21 | 0.138 | 0.482 |
|  | Base + SPEI03 | -372.76 | 0.138 | 0.485 |
|  | Base + SPEI12 | -372.24 | 0.138 | 0.483 |
|  | Base + SPEI03 + SPEI12 | -370.77 | 0.138 | 0.485 |

**Table S2: Model output for linear mixed models to predict monthly home range size and distance moved.** Model output is shown for univariate models per predictor and for the best-supported multivariate model. All models included individual ID as a random effect. Bold text indicates predictors that were significant in univariate but not in multivariate models. *N* = 1,025 elephant-year-month combinations.

|  |  | Univariate analysis | | | Multivariate analysis | | |
| --- | --- | --- | --- | --- | --- | --- | --- |
| Response | Predictor | Coefficient | Standard error | *p* value | Coefficient | Standard error | *p* value |
| Monthly home range | HII | -0.279 | 0.020 | *<*0.001 | -0.266 | 0.020 | *<*0.001 |
|  | **NDVI** | **0.058** | **0.015** | *<***0.001** | **0.011** | **0.018** | **0.543** |
|  | **Precipitation** | **0.076** | **0.015** | *<***0.001** | **0.016** | **0.017** | **0.356** |
|  | SPEI01 | 0.094 | 0.015 | *<*0.001 | 0.060 | 0.015 | *<*0.001 |
|  | Temperature | 0.099 | 0.015 | *<*0.001 | 0.101 | 0.015 | *<*0.001 |
|  |  |  |  |  |  |  |  |
| Monthly distance moved | HII | -0.071 | 0.010 | *<*0.001 | -0.079 | 0.009 | *<*0.001 |
|  | NDVI | -0.028 | 0.006 | *<*0.001 | -0.043 | 0.008 | *<*0.001 |
|  | Precipitation | 0.001 | 0.006 | 0.835 | 0.008 | 0.008 | 0.281 |
|  | SPEI01 | 0.016 | 0.007 | 0.014 | 0.020 | 0.007 | 0.003 |
|  | Temperature | 0.048 | 0.006 | *<*0.001 | 0.040 | 0.006 | *<*0.001 |

**Table S3: Model output for a resource selection function (RSF) with interactions between environmental covariates and SPEI01.** This model included elephant ID as a random effect. *N* = 57 elephants.

| Term | Coefficient | Standard error | *p* value |
| --- | --- | --- | --- |
| Intercept | -3.452 | 0.035 | <0.001 |
| Distance to inland water | -0.0793 | 0.0027 | <0.001 |
| SPEI01 | -0.0173 | 0.0017 | <0.001 |
| Population density | 0.0272 | 0.002 | <0.001 |
| Slope | 0.0118 | 0.0015 | <0.001 |
| Tree cover | -0.4673 | 0.0023 | <0.001 |
| Croplands | -0.0116 | 0.0015 | <0.001 |
| NDVI | 0.1739 | 0.002 | <0.001 |
| Precipitation | -0.0781 | 0.0022 | <0.001 |
| Distance to waterhole | 0.0251 | 0.0022 | <0.001 |
| SPEI01:Distance to inland water | 0.0074 | 0.0019 | <0.001 |
| SPEI01:Population density | 0.0092 | 0.0018 | <0.001 |
| SPEI01:Slope | 0.003 | 0.0016 | 0.058 |
| SPEI01:Tree cover | -0.0525 | 0.0019 | <0.001 |
| SPEI01:Croplands | 0.0016 | 0.0015 | 0.277 |
| SPEI01:NDVI | 0.0005 | 0.0021 | 0.818 |
| SPEI01:Precipitation | 0.0107 | 0.002 | <0.001 |
| SPEI01:Distance to waterhole | -0.0045 | 0.0017 | 0.01 |

**Table S4: Model output for a resource selection function (RSF) with interactions between environmental covariates and SPEI03.** This model included elephant ID as a random effect. *N* = 57 elephants.

| Term | Coefficient | Standard error | *p* value |
| --- | --- | --- | --- |
| Intercept | -3.4516 | 0.035 | <0.001 |
| Distance to inland water | -0.0723 | 0.0027 | <0.001 |
| SPEI03 | -0.0473 | 0.0018 | <0.001 |
| Population density | 0.0262 | 0.002 | <0.001 |
| Slope | 0.0141 | 0.0015 | <0.001 |
| Tree cover | -0.4692 | 0.0023 | <0.001 |
| Croplands | -0.0115 | 0.0015 | <0.001 |
| NDVI | 0.1835 | 0.002 | <0.001 |
| Precipitation | -0.074 | 0.002 | <0.001 |
| Distance to waterhole | 0.028 | 0.0022 | <0.001 |
| SPEI03:Distance to inland water | -0.0073 | 0.002 | <0.001 |
| SPEI03:Population density | 0.0002 | 0.0017 | 0.923 |
| SPEI03:slope | 0.0129 | 0.0015 | <0.001 |
| SPEI03:Tree cover | -0.0466 | 0.002 | <0.001 |
| SPEI03:Croplands | 0.0048 | 0.0015 | 0.001 |
| SPEI03:NDVI | -0.0019 | 0.0022 | 0.372 |
| SPEI03:Precipitation | 0.0133 | 0.002 | <0.001 |
| SPEI03:Distance to waterhole | -0.0102 | 0.0017 | <0.001 |

**Table S5: Model output for a resource selection function (RSF) with interactions between environmental covariates and SPEI12.** This model included elephant ID as a random effect. *N* = 57 elephants.

| Term | Coefficient | Standard error | *p* value |
| --- | --- | --- | --- |
| Intercept | -3.445 | 0.0355 | <0.001 |
| Distance to inland water | -0.0824 | 0.0027 | <0.001 |
| SPEI12 | -0.0084 | 0.0019 | <0.001 |
| Population density | 0.0244 | 0.002 | <0.001 |
| Slope | 0.0119 | 0.0015 | <0.001 |
| Tree cover | -0.4676 | 0.0023 | <0.001 |
| Croplands | -0.0118 | 0.0015 | <0.001 |
| NDVI | 0.1674 | 0.0019 | <0.001 |
| Precipitation | -0.0718 | 0.0018 | <0.001 |
| Distance to waterhole | 0.0269 | 0.0022 | <0.001 |
| SPEI12:Distance to inland water | -0.018 | 0.002 | <0.001 |
| SPEI12:Population density | -0.0072 | 0.002 | <0.001 |
| SPEI12:Slope | 0.0038 | 0.0017 | 0.026 |
| SPEI12:Tree cover | -0.0573 | 0.0024 | <0.001 |
| SPEI12:Croplands | -0.0066 | 0.0015 | <0.001 |
| SPEI12:NDVI | -0.0043 | 0.002 | 0.031 |
| SPEI12:Precipitation | 0.006 | 0.0018 | 0.001 |
| SPEI12:Distance to waterhole | -0.01 | 0.0018 | <0.001 |
